# A Novel Malaria Lateral Flow Assay for Detecting *Plasmodium falciparum* Lactate Dehydrogenase in Busia, Uganda

**DOI:** 10.4269/ajtmh.21-0956

**Published:** 2022-01-17

**Authors:** Christine M. Bachman, David M. Cate, Ben Grant, Stephen Burkot, Jerry Mulondo, Helen V. Hsieh, Martin Chamai, Bakar Odongo, Peter Olwoch, Mayimuna Nalubega, Harriet Ochokoru, Joseph Kasozi, John Ategeka, Kevin P. Nichols, Bernhard H. Weigl, Bryan Greenhouse

**Affiliations:** ^1^ Global Health Laboratories, Bellevue, Washington;; ^2^ Infectious Diseases Research Collaboration, Kampala, Uganda;; ^3^ Intellectual Ventures Laboratory, Bellevue, Washington;; ^4^ University of California at San Francisco, San Francisco, California

## Abstract

Rapid diagnostic tests (RDTs) for *Plasmodium falciparum* commonly detect histidine-rich protein 2 (HRP-2), but HRP-2 deletions are increasingly recognized. We evaluated a prototype test detecting parasite lactate dehydrogenase (pLDH) and compared it to commercially available RDTs at a health facility in Uganda, using quantitative polymerase chain reaction as a gold standard. The prototype pLDH test had a high sensitivity for infections with at least 100 parasites/µL (98%), comparable to HRP-2, and greater than an existing pLDH RDT (89%). Specificity for the prototype test was 99.5%, which is greater than the HRP-2 tests (93–95%). Therefore, the prototype pLDH test may be an attractive alternative malaria diagnostic.

## INTRODUCTION

In 2019, malaria cases numbered 229 million worldwide, an annual estimate that has remained nearly unchanged for the past 4 years.^1^ Rapid diagnostic tests (RDTs) play a central role in the diagnosis and control of malaria, especially in remote places where slide microscopy is not available. Malaria microscopy can also be a challenge because accuracy is affected by variability in the experience and training of microscopists and requires reliable access to quality microscopes. More sensitive nucleic acid-based tests such as quantitative polymerase chain reaction (qPCR) can detect lower density infections than microscopy or RDTs^2^; however, they are more expensive, have longer turnaround times, and are not normally available at the point of care because of the laboratory expertise, equipment, and reagents required.

Most currently available malaria RDTs detect histidine-rich protein 2 (HRP-2), parasite lactate dehydrogenase (pLDH), or aldolase as a marker of infection. HRP-2-based RDTs for *Plasmodium falciparum* malaria perform effectively in case management scenarios, detecting malaria infections at a threshold of approximately 100 parasites/µL, which is roughly equivalent to microscopy.^3^ RDTs using pLDH so far have demonstrated a lower sensitivity than HRP-2 tests for *P. falciparum*.^4^ Despite the good performance of HRP-2-based RDTs,^5^ HRP-2 is becoming less reliable as a biomarker of *P. falciparum *infection in some populations. The first problem is that HRP-2 gene deletions in parasite populations have become more prevalent, leading to false-negative test results. This issue has long been recognized in the Amazon of South America,^6^ and more research now demonstrates that deletions are also occurring in Africa^7^ and India,^8^ creating a need to identify alternative diagnostics. Also, HRP-2 can persist in the bloodstream after clearance of infection, generating false-positive test results.^9^ Although current RDTs based on pLDH are less sensitive than those based on HRP-2, pLDH is typically cleared from the bloodstream quickly and therefore is less likely to result in false positives resulting from persistence.

To overcome these limitations, Global Health Laboratories, alongside Access Bio, designed a more sensitive *P. falciparum *pLDH-detecting RDTs. The University of California at San Francisco and the Infectious Disease Research Collaboration evaluated clinical performance of the test in febrile patients presenting to a health facility in Uganda. The objective of this study was to evaluate the performance of the pLDH prototype test against other RDTs, including the best-in-class commercial pLDH test and the standard-of-care HRP-2 test. Microscopy, qPCR, and quantitative detection of pLDH using the Quansys platform were all used as references to compare sensitivity, specificity, and level of detection of the point-of-care tests.

## METHODS

We performed a prospective cross-sectional study of patients seeking care at the Masufu Hospital in Busia, Uganda. Patients eligible for inclusion were those age 0.5 to 75 years presenting to the clinic with suspected malaria based on a history of fever within the past 24 hours or objective fever (temperature > 38.0°C tympanic). We excluded patients with symptoms of complicated malaria or those who reported receiving any antimalarial medication in the previous month.

Study personnel conducted the informed consent discussion in the appropriate language. For children younger than 18 years of age, written informed consent was obtained from the parent/guardian. In addition, for children 8 to 17 years, assent was obtained.

For microscopy, thick blood smears were stained with 2% Giemsa stain. Parasite densities were calculated by counting the number of asexual parasites per 200 leukocytes, assuming a leukocyte count of 8,000 leukocytes/µL.^10^ A blood smear was considered negative when the examination of 100 high-power fields did not reveal asexual parasites. For the study, final microscopy results were read by two independent microscopists, and a third reviewer settled discrepant readings.

For assessment of the RDT results, the two independent operators assessed each test for the presence or absence of test and control lines. The pLDH prototype used 5 µL of whole blood and 75 µL of buffer, with a 20-minute read time. Instructions for use were followed for all commercial tests. These tests included the CareStart™ Malaria Pf (HRP2) Ag RDT (model RMOM-03091; Access Bio, Somerset, NJ) (standard of care in Uganda) and the SD Bioline Malaria Ag Pf (HRP2/pLDH) (model 05FK90; Abbott Laboratories, Abbott Park, IL).

For qPCR, DNA was extracted from 200 µL of whole blood using the PureLink Genomic DNA Mini Kit (Invitrogen). Parasitemia was quantified using an ultrasensitive *var* gene acidic terminal sequence (*var*ATS) qPCR assay with a lower limit of detection of 0.05 parasites/µL.^11^ Quantification was accomplished by regression of cycle thresholds against a standard curve run on each plate. Samples for the standard curves were derived from ring-synchronized cultured parasites spiked into human whole blood at concentrations ranging from 0.05 parasites/µL to 10,000 parasites parasites/µL and extracted concomitantly with study samples along with negative controls.

The Q-Plex™ Human Malaria Array kit (Quansys, West Logan, UT) was used to measure the concentration of HRP-2 and pLDH in frozen whole blood samples per manufacturer instructions. Frozen blood samples were diluted 1:4 and 1:100 from the stock. Sample concentrations were determined using Quansys-provided Q-View software.

Sample size was determined by the prevalence of malaria and to ensure that the sensitivity and specificity of the pLDH test was within 20% margin of error of expected values (80% power, 0.05 significance level).^12^ Exact binomial confidence limits were calculated for test sensitivity, specificity, and positive and negative predictive value by comparing a visually read test result to microscopy, qPCR, and Quansys. A sample was considered positive by qPCR if cycle thresholds were at or less than the lowest positive control on the standard curve. Analysis was performed using R (version 3.6.0).^13^

## RESULTS AND DISCUSSION

A total of 395 individuals participated in this study (Supplemental Table 1). Eight percent of participants had an objective fever measured at the time of presentation to the clinic. Approximately half of participants had *P. falciparum *detected by qPCR, but only 11% had parasite densities of at least 1,000 parasites/µL as measured by qPCR.

pLDH concentration in the blood correlated strongly with estimates of parasite density from qPCR (Spearman’s rho = 0.88), but there was still a variation of ≈30 fold in pLDH concentration for a given parasite density (Supplemental Figure 1). The majority of qPCR-positive samples were also positive by pLDH, but more than half of the qPCR-negative samples had pLDH detected at some level (Supplemental Table 2). These discordant samples tended to have very low concentrations of pLDH detected (Supplemental Figure 1), and all except one were negative by microscopy, suggesting they may represent false-positive tests as a result of persistent pLDH or as a result of the Quansys assay from cross-contamination or matrix effects from whole blood. Microscopy and qPCR had an expected relationship, with microscopy detecting nearly all infections with qPCR parasite densities greater than 100 parasites/µL, but with very limited sensitivity at less than 10 parasites/µL (Supplemental Figure 1).

We evaluated the performance of the RDTs compared with these reference standards. The pLDH prototype RDT had a sensitivity of 98% for infections with at least 100 parasites/µL, comparable to both HRP-2 RDTs and significantly greater than the commercial pLDH-based test, which had a sensitivity of 89% for these infections (*P* = 0.03, Table 1). For low-density infections near the limit of detection by microscopy and standard RDTs (20–200 parasites/µL), sensitivity for the pLDH prototype RDT remained reasonably high at 80%, compared with 63% for the existing pLDH-based test and 87% to 89% for the HRP-2-based tests. Although sensitivity for all tests dropped off for lower density infections, the pLDH prototype RDT remained substantially more sensitive than the existing pLDH test and less sensitive than HRP-2 tests. Specificity for both pLDH based tests was excellent (99.5%) and greater than the HRP-2-based tests (93–95%).

**Table1:** Visual sensitivity and specificity for the pLDH prototype and two commercial HRP2 and Pf LDH tests as compared to qPCR

Statistic	Parasite density (no. of parasites/µL blood)	pLDH prototype, LDH TL	SD Bioline malaria Ag Pf (HRP-2/pLDH)		CareStart™ malaria Pf Ag (HRP-2)
			LDH TL-1	HRP-2 TL-2	HRP-2 TL
Sensitivity	Overall	59% 52–65, 119/203	34%, 28–41, 70/203	69%, 63–76, 141/203	73%, 67–79, 149/203
	≥ 0.05 < 20	31%, 22–40, 34/110	4%, 1–9, 4/110	48%, 39–58, 53/110	55%, 45–64, 60/110
	≥ 20 < 100	75%, 55–89, 21/28	29%, 13–49, 8/28	89%, 72–98, 25/28	89%, 72–98, 25/28
	≥ 100 < 200	90%, 55–100, 9/10	40%, 12–74, 4/10	80%, 44–97, 8/10	90%, 55–100, 9/10
	≥ 200 < 1,000	100%, 72–100, 11/11	91%, 59–100, 10/11	100%, 72–100, 11/11	100%, 72–100, 11/11
	≥ 1,000	100%, 92–100, 44/44	100%, 92–100, 44/44	100%, 92–100, 44/44	100%, 92–100, 44/44
Specificity	Overall	99%, 97–100, 191/192	99%, 97–100, 191/192	95%, 91–97, 182/192	93%, 89–96, 179/192

HRP-2 = histidine-rich protein 2; LDH = lactate dehydrogenase; pLDH = parasite lactate dehydrogenase; TL = test line.

**Figure 1.**
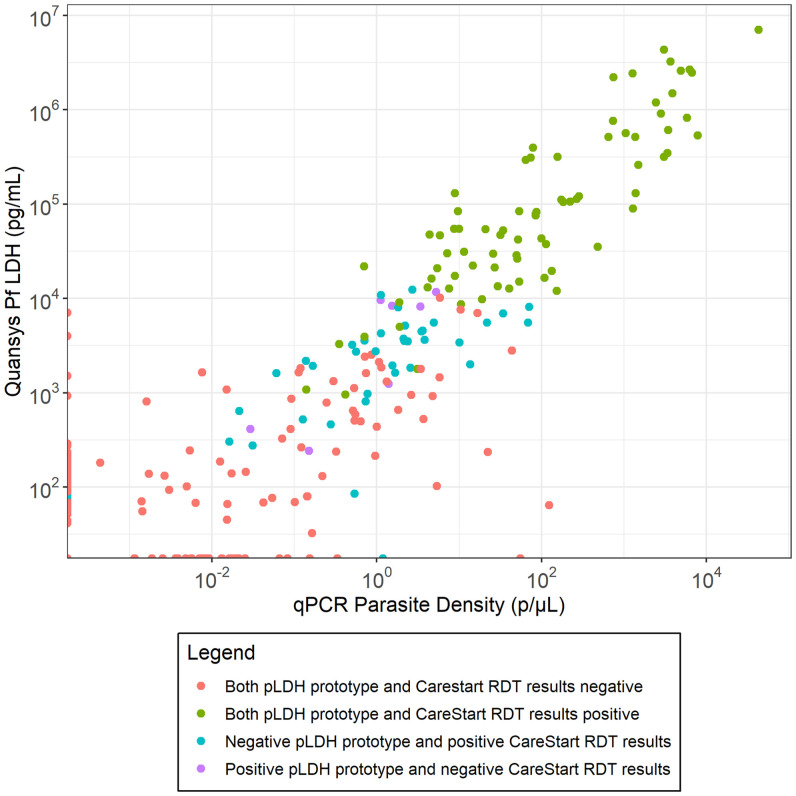
Visual read results for pLDH prototype and CareStart HRP-2 RDTs as a function of qPCR parasite density and pLDH concentration. p = parasites; Pf LDH = *Plasmodium falciparum* lactate dehydrogenase; pLDH = parasite lactate dehydrogenase; qPCR = quantitative polymerase chain reaction; RDT = rapid diagnostic test.

We compared RDT results to pLDH concentration and parasite densities obtained from qPCR (Figure 1). Detectability by the pLDH prototype RDT tracked closely with pLDH concentration. Those detected by the CareStart^TM^ HRP-2 RDT, but not the pLDH prototype, had a very consistent relationship with pLDH concentration despite variation in parasite densities as measured by qPCR.

The prototype pLDH RDT evaluated here demonstrated excellent sensitivity for infections likely to cause illness and generally detectable by microscopy, comparable to existing HRP-2-based RDTs and better than the best commercial pLDH-based test. Although the sensitivity of this new test appears sufficient for the diagnosis of malaria in this initial study, sensitivity in low-density infections was less than that of HRP-2-based RDTs, with potential implications for screening asymptomatic populations for parasitemia. On the other hand, specificity of the pLDH RDT was much greater than HRP-2-based RDTs, as might be expected given the shorter half-life of pLDH versus HRP-2 in the blood. Quantitative measurement of pLDH confirmed that the prototype RDTs are performing as expected, with pLDH levels matching visual results.

There are limitations to this study that may not make it generalizable. Malaria transmission was very high in the Busia District of Uganda when this evaluation occurred, potentially resulting in a larger proportion of low-density infections resulting from acquired immunity and/or many of these infections being “older” with lower parasite density and not necessarily being the cause of the acute illness. However, this distribution of parasite density allowed for the evaluation of sensitivity near the limit of detection.

Evaluating this test more widely, particularly in settings were HRP-2 deletion is present, would demonstrate its use case more effectively and should be pursued given the lack of suitable alternative diagnostics.

## Supplemental Material


Supplemental materials
