# Supplementary material for: A Novel Malaria Lateral Flow Assay for Detecting Plasmodium falciparum Lactate Dehydrogenase in Busia, Uganda
Source: Am J Trop Med Hyg. 2022 Jan 17;106(3):850–2. doi: 10.4269/ajtmh.21-0956 (PMC8922488; doi:10.4269/ajtmh.21-0956)
Supplement: Supplementary file 1 [file tpmd210956.SD1.pdf]

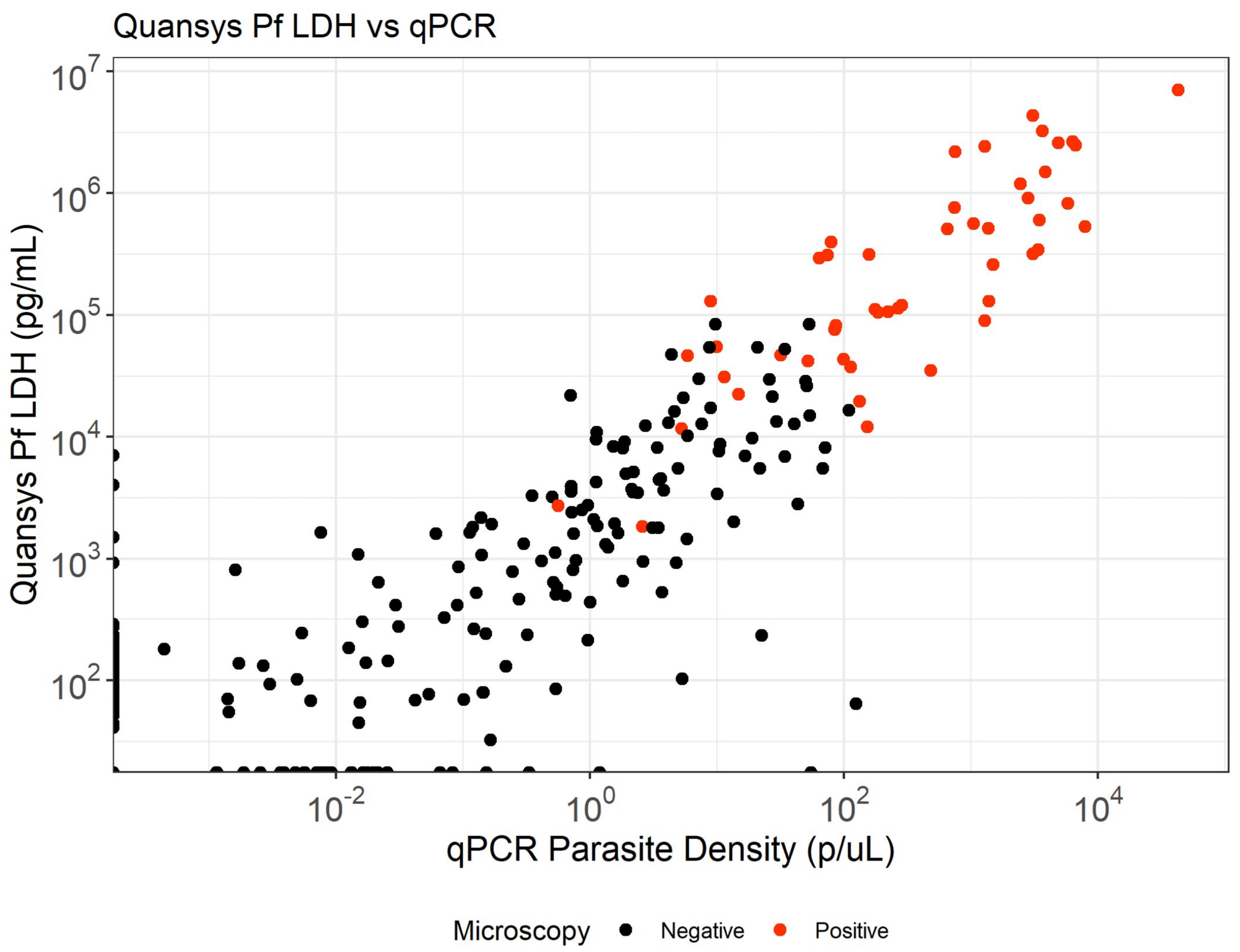

Quansys Pf LDH vs qPCR
Quansys Pf LDH (pg/mL)
10^7
10^6
10^5
10^4
10^3
10^2
10^{-2}
10^0
10^2
10^4
qPCR Parasite Density (p/uL)
Microscopy
Negative
Positive

Supplemental Table 1 | Study Participants

Category	
Number of participants	395
Identifying as female, n (%)	291 (74%)
Median age in years (IQR)	26 (8.5 – 41)
Tympanic temperature >38 °C, n (%)	30 (8%)
Positive by qPCR, n (%)	203 (51%)
Median parasite density of qPCR+, parasites/μL (IQR)	10.0 (1.1 – 350.7)

Supplemental Table 2 | qPCR

qPCR Positive		Microscopy Positive	Microscopy Negative
203	Quansys Pf LDH Positive	82	115
	Quansys Pf LDH Negative	0	6
qPCR Negative		Microscopy Positive	Microscopy Negative
192	Quansys Pf LDH Positive	1	109
	Quansys Pf LDH Negative	0	82
